# Associations of dietary flavones, particularly apigenin and luteolin, with phenotypic age acceleration: A cross-sectional study using NHANES data

**DOI:** 10.1097/MD.0000000000046520

**Published:** 2025-12-12

**Authors:** Xiaoqiao Wang, Chang Liu, Guixia Li, Shuyan Tian, Wujian Peng, Peijia Liu

**Affiliations:** a Department of Nephrology, Shenzhen Third People’s Hospital, The Second Affiliated Hospital of Southern University of Science and Technology, Shenzhen, Guangdong Province, China.

**Keywords:** apigenin, biological aging, epidemiology, flavone, luteolin, NHANES, nutrition

## Abstract

Specific flavone subclasses, particularly apigenin and luteolin, exhibit potent anti-aging properties mediated by their anti-inflammatory and antioxidant activities. However, epidemiological evidence relating these bioactive compounds to phenotypic age acceleration (PhenoAgeAccel) remains limited. We analyzed data from 10,789 US adults participating in the National Health and Nutrition Examination Survey 2007–2010 and 2017–2018 cycles. PhenoAgeAccel was calculated as the residuals from regressing phenotypic age (based on inflammatory and metabolic biomarkers) on chronological age, where positive values indicated accelerated aging. Intakes of total flavones, apigenin, and luteolin were quantified using 24-h dietary recalls. Weighted multivariable logistic regression models were used to assess associations between flavone intakes and PhenageAccel, with comprehensive adjustments for potential confounders. Restricted cubic spline models were employed to evaluate non-linear relationships. Higher total flavone intake was associated with a dose-dependent decrease in PhenoAgeAccel (*P*-trend < .001). In fully adjusted models, each log-unit increase (equivalent to 2.7-fold higher intake) in flavone intake corresponded to a 9.6% reduction in the odds of PhenoAgeAccel (odds ratio [OR] = 0.904, 95% confidence interval: 0.859–0.953). Similar inverse associations were observed for apigenin (Q4 vs Q1: OR = 0.647, *P* = .002) and luteolin (Q4 vs Q1: OR = 0.736, *P* = .011). Significant non-linear dose-response relationships were observed for all flavones (*P*-nonlinearity < 0.001). Subgroup analyses unveiled consistent associations across age, sex, and cardiometabolic status (all *P*-interaction > 0.05). In this nationally representative sample, higher dietary intakes of flavones, particularly apigenin and luteolin, were strongly associated with reduced PhenoAgeAccel. These findings suggest the potential role of flavones as modifiable dietary factors for healthy aging.

## 1. Introduction

Aging is a progressive, time-dependent decline in organismal homeostasis, marked by the cumulative dysfunction of physiological systems. By 2050, approximately 2 billion individuals, about 20% of the global population, will be aged ≥ 65 years, underscoring the urgent need to address aging as a public health priority.^[1]^ Unlike chronological age, which merely reflects the passage of time, biological aging captures individual variability in health trajectories. Phenotypic age (PhenoAge) offers a quantifiable measure of biological aging, integrating biomarkers of multi-system decline, such as albumin, creatinine, and inflammatory cytokines.^[2,3]^ We focused on PhenoAgeAccel rather than DNA methylation clocks or telomere length, as it not only integrates clinically accessible biomarkers but has also been robustly validated in large cohorts. As a well-established indicator of accelerated aging, PhenoAgeAccel demonstrates consistent associations with key clinical outcomes including physical frailty, cognitive decline, and premature mortality across diverse populations.^[4–13]^ Identifying modifiable factors that can decelerate biological aging is thus critical for promoting healthy longevity.

Among potential interventions, dietary flavonoids have garnered growing interest for their capacity to modulate biological aging.^[14–16]^ Flavones, a subclass of flavonoids found in parsley, celery, chamomile, citrus fruits, and various medicinal plants, are known for their potent antioxidant, anti-inflammatory, anticancer, and metabolic regulatory effects.^[17–21]^ Emerging evidence suggests that flavones may exert some of the most powerful anti-aging effects within the flavonoid family, acting on key aging pathways through their antioxidant and anti-inflammatory properties.^[22]^ Specifically, luteolin, a prominent flavone, has demonstrated anti-aging effects, particularly on the skin by modulating key signaling pathways such as nuclear factor kappa-light-chain-enhancer of activated B cells (NF-κB), Janus kinase-signal transducer and activator of transcription (JAK-STAT), and Toll-like receptors (TLR).^[23]^ It suppresses pro-inflammatory cytokines, like interleukin (IL)-6, and tumor necrosis factor-alpha (TNF-α), while enhancing the body’s antioxidant defenses.^[23]^ Apigenin, another dietary flavone, has shown strong neuroprotective potential through its regulation of neuroinflammation, oxidative stress, and cellular senescence pathways.^[24]^ It may also boost levels of nicotinamide adenine dinucleotide (NAD⁺), modulate inflammation and neural signaling, and improve aging, sleep, cognition, and lifespan outcomes.^[25]^ These mechanisms highlight flavones as promising agents for modulating PhenoAgeAccel and biological aging more broadly.

Despite these mechanistic insights, population-level evidence on the association between dietary flavone intake and PhenoAgeAccel remains sparse. The current study seeks to address this gap by utilizing data from the National Health and Nutrition Examination Survey (NHANES), a nationally representative US survey with detailed dietary and biomarker data. We hypothesize that higher dietary intakes of total flavones and key subclasses (luteolin and apigenin) are associated with lower PhenoAgeAccel risk. Our findings aim to provide novel evidence for the role of flavones as modifiable dietary components in promoting healthy biological aging.

## 2. Methods

### 2.1. Study design and study population

The NHANES database is a publicly accessible, nationally representative database that collects data biennially through a complex, multistage, stratified sampling design. It covers extensive information, including demographics, dietary intake, physical examinations, laboratory test results, and health-related survey responses. NHANES ensures the representativeness of the US population by applying specific sampling weights to adjust for oversampling and nonresponse biases.^[26]^

**Figure 1.**
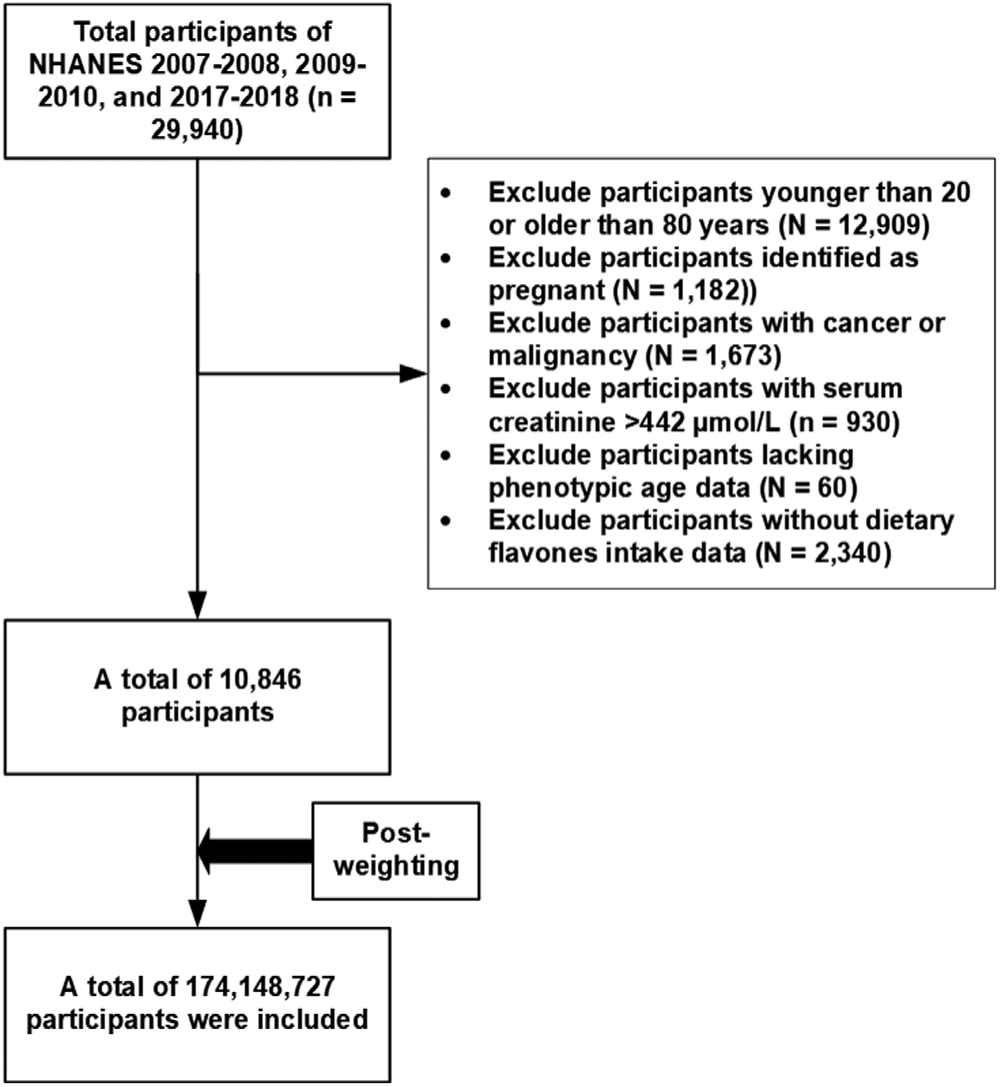
Participants selection.

This cross-sectional analysis utilized flavonoid intake data from the USDA Flavonoid Values for the USDA Food Codes Database (FNDDS version 4.1 and 5.0). We analyzed 3 NHANES cycles (2007–2008, 2009–2010, 2017–2018). Initial pooled data included 29,940 participants. The exclusion criteria were as follows: age < 20 or ≥ 80 years; pregnancy; severe renal impairment (serum creatinine > 442 μmol/L); missing flavone intake data; incomplete phenotypic age biomarkers; or cancer or malignancy history. After exclusions, 10,846 participants were analyzed. Using NHANES sampling weights, the final analytical sample consisted of 174,148,727 individuals, which constituted a nationally representative sample of US adults (Fig. 1).

Each participant in NHANES provided written informed consent, and the collection of survey data was approved by the Research Ethics Review Board of the NCHS. The data utilized for these analyses have been fully de-identified and made publicly available. This study was a secondary analysis of a public dataset and did not require ethics approval again.

### 2.2. Dietary intake of flavones and their subclasses in US adults

The dietary intake of total flavones and their subclasses (i.e., apigenin and luteolin) was estimated by using a standardized methodology developed by the USDA.^[27]^ Trained interviewers conducted 2 nonconsecutive 24-h dietary recalls via the Automated Multiple-Pass Method: the first in-person at an NHANES Mobile Examination Center and the second via telephone after 3–10 days.^[28]^ Total flavone intake (mg/day) was calculated by summing the product of each food’s flavone content (from FNDDS) and its daily consumption amount (g/day), which were then averaged across 2 recalls. The additional methodological details are described elsewhere.^[27,29,30]^

### 2.3. Study outcomes

Phenotypic age was calculated based on the equation proposed by Levine et al. (2018), which included 9 clinical biomarkers (e.g., albumin, creatinine), with full computational procedures, as detailed in Method S1 of Supplementary File 1, Supplemental Digital Content, https://links.lww.com/MD/Q885 (*2*). PhenoAgeAccel was defined as the residuals from a linear regression model wherein the phenotypic age was regressed on chronological age. Positive residuals (PhenoAgeAccel > 0) indicated accelerated biological aging relative to the chronological age.

### 2.4. Covariates

The covariates incorporated in this study encompassed demographic characteristics, lifestyle factors, and clinical parameters. The demographic variables included age (years), sex (male or female), and race/ethnicity (Mexican American, non-Hispanic White, non-Hispanic Black, and others, including multiracial individuals). The educational attainment was categorized as middle-school or lower versus high-school or higher. Economic status was assessed using the poverty income ratio (PIR), with a PIR < 1 indicating poverty and a PIR ≥ 1 representing nonpoverty. The marital status was categorized into 2 groups: “Marriage or Partnered,” which includes individuals who were legally married or in a long-term cohabiting partnership, and “Other,” which encompassed individuals who were single, divorced, widowed, or separated. The lifestyle-related variables included smoking status, alcohol consumption, physical activity, and dietary energy intake. The smoking status was categorized as former smokers (those who have smoked ≥ 100 cigarettes in their lifetime and have quit), never smokers (those who have smoked < 100 cigarettes in their lifetime), and current smokers (those who have smoked ≥ 100 cigarettes in their lifetime and are currently smoking). The alcohol consumption status was categorized as never drinker (those who have not consumed alcohol in the past 12 months and have no history of drinking), former drinker (those who have consumed alcohol in the past, but have not drunk in the past 12 months and have consumed ≥ 12 drinks in their lifetime), and current drinker (those who have consumed alcohol in the past 12 months). The physical activity levels were assessed using the metabolic equivalent of task (MET)-min per week and categorized into the following 3 groups: insufficient (<600 MET-min/week, <150 minutes moderate-intensity), active (600–1200 MET-min/week, 150–300 minutes moderate-intensity), and highly active (>1200 MET-min/week, >300 minutes moderate-intensity).^[31]^ The dietary energy intake was calculated as the average total kilocalories consumed over two 24-h dietary recalls. Body mass index (BMI) was computed as weight in kilograms divided by the square of height in meters (kg/m²). Hypertension was defined as a systolic blood pressure of ≥ 140 mm Hg, diastolic blood pressure of ≥ 90 mm Hg, use of antihypertensive medications, or a clinical diagnosis of hypertension.^[32]^ Diabetes was defined by the presence of one or more of the following conditions: glycated hemoglobin > 6.5%, fasting plasma glucose > 7.0 mmol/L, random plasma glucose > 11.1 mmol/L, 2-h postprandial glucose > 11.1 mmol/L, use of antidiabetic medication, or a clinical diagnosis of diabetes.^[33]^ Coronary vascular disease includes coronary heart disease (CHD), congestive heart failure, myocardial infarction (MI), angina, and stroke. The classification of depression severity was based on the PHQ-9 score, where a score of ≥ 10 indicated clinically significant depression, and a score of < 10 indicated no significant depression.^[34]^

### 2.5. Statistical analysis

The NHANES dataset was collected by using a complex, multistage stratified sampling approach. All data were weighted before statistical analysis to ensure the national representativeness of the US adult population. Continuous variables were summarized as means with 95% confidence intervals (CIs) and compared between the PhenoAgeAccel and non-PhenoAgeAccel groups using the Wilcoxon rank-sum test. Categorical variables were expressed as percentages with 95% CIs and then compared using the Chi-square test. The dietary intake of total flavones and their major subclasses (apigenin and luteolin) was assessed using 24-hour dietary recall and analyzed as both quartiles (Q1–Q4) for categorical analyses and log-transformed for continuous analyses. Weighted multivariable logistic regression models were employed to assess the association between flavone intake and PhenoAgeAccel, with trend tests applied across quartiles. Restricted cubic spline (RCS) models with 3 knots placed at the 10th, 50th, and 90th percentiles of exposure distributions were employed to evaluate potential nonlinear dose–response relationships between flavone intake and PhenoAgeAccel. Stratified analyses were performed to examine the potential effect modification by demographic (such as age group, sex, and ethnicity), socioeconomic (such as poverty-income ratio, education, and marital status), lifestyle (such as total energy intake, physical activity, smoking, and alcohol use), and clinical characteristics (such as BMI, depression, and cardiometabolic diseases). Interaction tests were performed to assess heterogeneity in the associations across subgroups. In addition, the associations between individual flavone subclasses, apigenin and luteolin, and PhenoAgeAccel were examined using weighted multivariable logistic regression. To assess the potential synergistic effects, an interaction term between apigenin and luteolin intake was added to the regression model to determine whether their combined intake had a greater-than-additive association with PhenoAgeAccel. Sensitivity analyses were conducted by including participants with cancer, pregnancy, and severe renal dysfunction (serum creatinine > 442 μmol/L) to evaluate the robustness of the findings. All statistical analyses were performed using R software (version 4.3.0, University of Auckland, NZ), with a 2-sided *P* < .05 to indicate statistical significance.

## 3. Results

### 3.1. Characteristics of the population

As show in Table 1, in this nationally representative sample of US adults, individuals with PhenoAgeAccel (39.1%) displayed distinct demographic, clinical, and lifestyle characteristics compared to those without PhenoAgeAccel (60.9%). The PhenoAgeAccel group included a higher proportion of males (59.8% vs 48.1%), had a younger mean age (46.5 vs 47.8 years; *P *= .03), elevated BMI (31.5 vs 27.5 kg/m²; *P *< .001), and a greater percentage of non-Hispanic Black individuals (15.6% *vs* 8.1%). Socioeconomic disparities were also evident. The PhenoAgeAccel group had lower educational attainment (high school or more: 20.8% vs 16.3%), a higher prevalence of poverty (PIR < 1: 17.5% vs 11.3%), and a lower proportion of married/partnered individuals (57.7% vs 66.9%) (all *P *< .001). Significant differences in lifestyle factors, including smoking status and physical activity, were also observed (all *P *< .05). Notably, individuals in the PhenoAgeAccel group had significantly lower intakes of total dietary flavones (0.80 vs 1.03 mg/day), apigenin (0.17 vs 0.27 mg), and luteolin (0.63 vs 0.76 mg) (all *P* ≤ .01). Cardiometabolic conditions were more prevalent in the PhenoAgeAccel group, including hypertension (44.6% vs 32.4%), diabetes (24.5% vs 6.7%), and cardiovascular disease (11.3% vs 6.3%) (all *P* < .001). Furthermore, clinically significant depression was more common (9.9% vs 5.9%; *P *< .001).

**Table 1:** Baseline characteristics of US adults by PhenoAgeAccel.

Variables	Overall (100%)	PhenoAgeAccel (39.1%)	Non-PhenoAgeAccel (60.9%)	*P* value
Sex, %				<.001
Female	47.3 (44.0,50.7)	40.2 (38.0, 42.4)	51.9 (50.2, 53.6)	
Male	52.7 (49.3, 56.0)	59.8 (57.6, 62.0)	48.1 (46.4, 49.8)	
Age (years old)	47.3 (46.7, 48.0)	46.5 (45.5, 47.6)	47.8 (47.1, 48.5)	.03
BMI (kg/m^2^)	29.1 (28.8, 29.3)	31.5 (31.2, 31.9)	27.5 (27.2, 27.8)	<.001
Ethnicity, %				<.001
Non-Hispanic Black	11.1 (9.4, 12.7)	15.6 (13.1, 18.1)	8.1 (6.5, 9.8)	
Mexican American	8.8 (6.9, 10.7)	10.0 (7.6, 12.3)	8.1 (6.0, 10.1)	
Other racial groups	12.9 (11.1, 14.8)	13.4 (11.3, 15.6)	12.6 (10.3, 14.9)	
Non-Hispanic White	67.2 (60.1, 74.3)	61.0 (57.1, 65.0)	71.2 (67.2, 75.2)	
Education levels, %				<.001
High school or more	74.7 (69.5, 79.9)	79.2 (77.5, 80.9)	83.7 (81.9, 85.5)	
Middle school or lower	16.4 (14.7, 18.0)	20.8 (19.1, 22.5)	16.3 (14.5, 18.1)	
PIR, %				<.001
≥ 1	80.2 (74.4, 85.9)	82.5 (80.8, 84.2)	88.7 (87.1, 90.2)	
< 1	12.8 (11.5, 14.0)	17.5 (15.8, 19.2)	11.3 (9.8, 12.9)	
Marital status, %				<.001
Married or partnered	63.3 (58.5, 68.1)	57.7 (54.6, 60.8)	66.9 (64.8, 69.1)	
Others	36.7 (34.1, 39.3)	42.3 (39.2, 45.4)	33.1 (30.9, 35.2)	
Smoking status, %				<.001
Former	24.7 (22.6, 26.7)	23.4 (21.4, 25.5)	25.5 (23.6, 27.4)	
Never	55.4 (51.5, 59.3)	50.6 (47.8, 53.3)	58.6 (55.8, 61.3)	
Now	19.9 (17.8, 22.0)	26.0 (23.6, 28.4)	16.0 (14.2, 17.8)	
Drinking status, %				.39
Former	10.6 (9.1, 12.1)	12.1 (10.4, 13.8)	11.5 (9.9, 13.1)	
Never	9.3 (8.2, 10.4)	9.5 (8.2, 10.9)	10.8 (9.3, 12.3)	
Now	70.6 (65.6, 75.7)	78.4 (76.2, 80.5)	77.8 (75.5, 80.0)	
Physical activity status, %				.02
Insufficient	16.2 (14.5, 17.9)	15.7 (13.8, 17.7)	16.5 (14.8, 18.2)	
Active	14.4 (13.2, 15.6)	12.7 (11.3, 14.1)	15.4 (14.4, 16.5)	
Highly active	69.4 (64.9, 74.0)	71.6 (69.4, 73.8)	68.1 (66.3, 69.9)	
Energy intake (kcal/d)	2180 (2147, 2212)	2188 (2143, 2233)	2174 (2127, 2221)	.7
Flavone (mg)	0.94 (0.87, 1.00)	0.80 (0.73, 0.86)	1.03 (0.93, 1.13)	<.001
Apigenin (mg)	0.23 (0.19, 0.27)	0.17 (0.15, 0.19)	0.27 (0.20, 0.34)	.01
Luteolin (mg)	0.71 (0.67, 0.75)	0.63 (0.57, 0.69)	0.76 (0.70, 0.81)	.003
Depression status, %				<.001
No significant depression	92.6 (86.7, 98.4)	90.1 (88.7, 91.5)	94.1 (93.0, 95.2)	
Clinically significant depression	7.4 (6.5, 8.4)	9.9 (8.5, 11.3)	5.9 (4.8, 7.0)	
CVD, %				<.001
No	91.7 (85.9, 97.5)	88.7 (86.9, 90.4)	93.7 (92.8, 94.6)	
Yes	8.3 (7.2, 9.3)	11.3 (9.6, 13.1)	6.3 (5.4, 7.2)	
Hypertension, %				<.001
No	62.8 (58.8, 66.9)	55.4 (52.4, 58.5)	67.6 (65.8, 69.4)	
Yes	37.2 (33.9, 40.5)	44.6 (41.5, 47.6)	32.4 (30.6, 34.2)	
Diabetes, %				<.001
No	86.3 (80.7, 92.0)	75.5 (73.6, 77.4)	93.3 (92.6, 94.1)	
Yes	13.7 (12.6, 14.7)	24.5 (22.6, 26.4)	6.7 (5.9, 7.4)	

Data are presented as mean for continuous variables or proportions for categorical variables with adjusted 95% confidence interval.

BMI = body mass index, CVD = cardiovascular disease, PhenoAgeAccel = phenotypic age acceleration, PIR = poverty income ratio.

### 3.2. Association between flavone intake and PhenoAgeAccel

**Figure 2.**
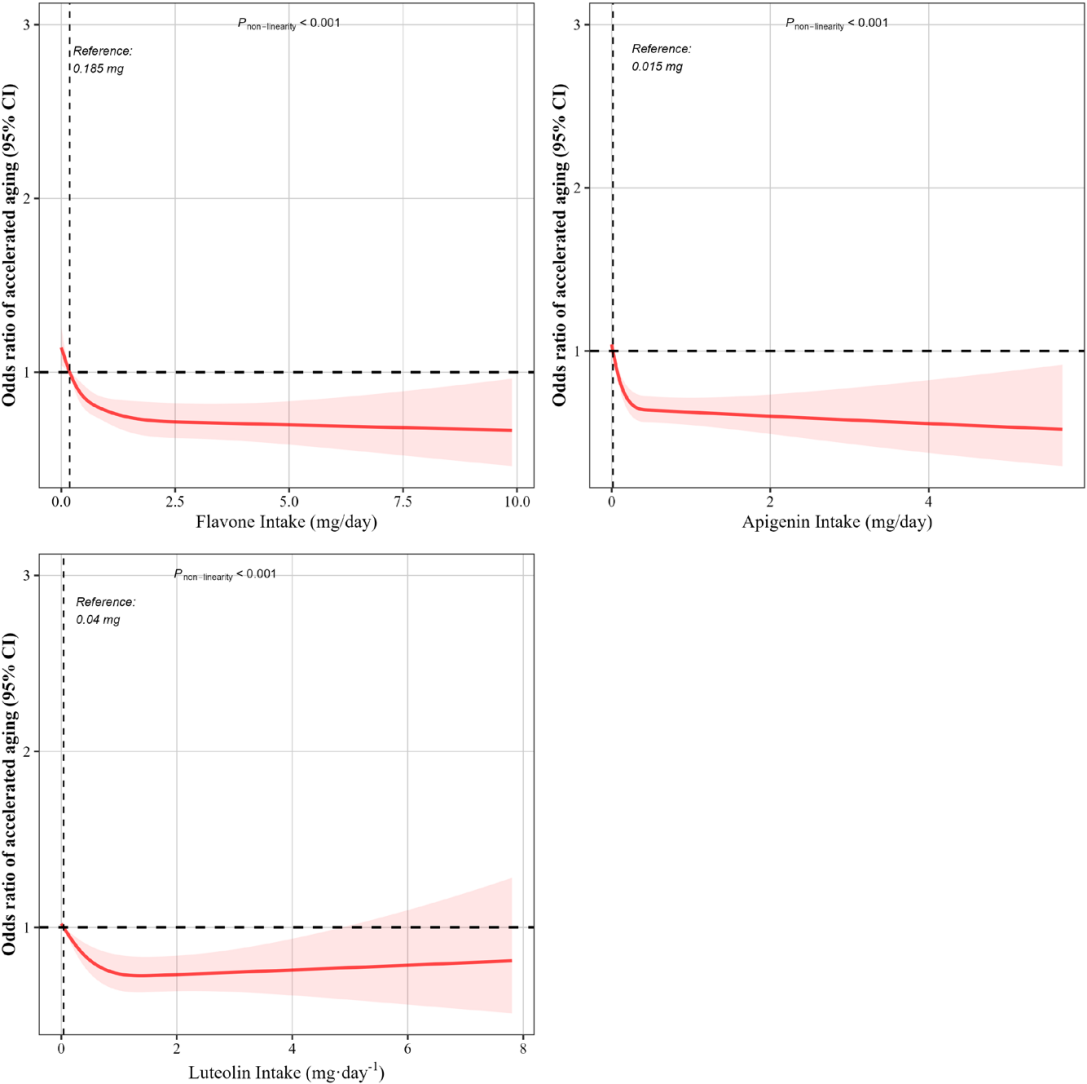
The non-linear trend between the intake of dietary intake of flavones and PhenoAgeAccel using a restricted cubic spline. Data are presented as OR (95%CI) (y-axis) and level of flavones (mg per 24 h) after adjusted for age, sex, body mass index, ethnicity, poverty status, education status, physical activity status, marital status, smoking status, alcohol consumption, energy intake, depression, cardiovascular disease, hypertension, and diabetes. OR = odds ratio, CI = confidence interval, PhenoAgeAccel = phenotypic age acceleration.

As shown in Table 2, higher dietary flavone intake was strongly and inversely associated with PhenoAgeAccel in a dose-dependent manner across all analytical models. In the fully adjusted model (Model 3), each log-unit increase (equivalent to 2.7-fold higher intake) in flavone intake was associated with a 9.6% reduction in PhenoAgeAccel (*β* = 0.904; 95% CI): 0.859–0.953; *P* < .001). When flavone intake was stratified by quartiles, a clear inverse trend was observed. Participants in the highest quartile (Q4) had significantly lower PhenoAgeAccel than those in the lowest quartile (Q1), with *β* estimates of 0.505 (95% CI: 0.404–0.632; *P* < .001), 0.502 (95% CI: 0.402–0.627; *P* < .001), and 0.681 (95% CI: 0.537–0.863; *P* = .003) in Models 1, 2, and 3, respectively. The trend across quartiles remained significant in all models (*P* for trend < .001), suggesting a robust inverse relationship even after adjusting for a wide range of potential confounders including demographic, socioeconomic, lifestyle, and health-related variables. Sensitivity analyses excluding participants with cancer, pregnancy, and severe renal dysfunction (serum creatinine > 442 μmol/L) confirmed the robustness of our primary findings, as the fully adjusted model (Model 3) continued to show significant inverse associations between higher dietary flavone intake and PhenoAgeAccel (continuous: *β* = 0.912, 95%CI 0.866–0.961, *P* = .001; Q4 vs Q1: OR = 0.674, 95%CI 0.538–0.846, *P* = .001) with a persistent dose-response relationship (*P* for trend < 0.001) after comprehensive adjustment for sociodemographic, lifestyle, and comorbidity factors (Table S1, Supplemental Digital Content, https://links.lww.com/MD/Q885). Furthermore, RCS analysis revealed a significant non-linear dose–response relationship between flavone intake and PhenoAgeAccel (*P* for non-linearity < .001) (Fig. 2).

**Table 2:** Association of dietary flavones intake with PhenoAgeAccel among US adults.

Flavones	Model 1	*P* value		Model 2	*P* value		Model 3	*P* value
	β (95% CI)			β (95%CI)			β (95%CI)	
Continuous	0.855 (0.817, 0.896)	<.001	Continuous	0.857 (0.817, 0.898)	< .001	Continuous	0.904 (0.859, 0.953)	<.001
Categories			Categories			Categories		
Q1	Ref		Q1	Ref		Q1	ref	
Q2	0.723 (0.624, 0.837)	<.001	Q2	0.748 (0.642, 0.871)	< .001	Q2	0.882 (0.710, 1.097)	<.001
Q3	0.590 (0.487, 0.717)	<.001	Q3	0.602 (0.497, 0.729)	< .001	Q3	0.755 (0.614, 0.930)	.01
Q4	0.505 (0.404, 0.632)	<.001	Q4	0.502 (0.402, 0.627)	< .001	Q4	0.681 (0.537, 0.863)	.003
*P* for trend	<.001			<.001			<.001	

Flavones intake was log-transformed and analyzed as a continuous variable in a regression model.

Model 1 did not include any covariate. Model 2 was adjusted for age and sex. Model 3 was adjusted for age, sex, body mass index, ethnicity, poverty status, education status, physical activity status, marital status, smoking status, alcohol consumption, energy intake, depression, cardiovascular disease, hypertension, and diabetes.

CI = confidence interval, PhenoAgeAccel = phenotypic age acceleration, Q1–Q4, = quartiles 1–4.

### 3.3. Subgroup analysis for the association between flavone intake and PhenoAgeAccel

**Figure 3.**
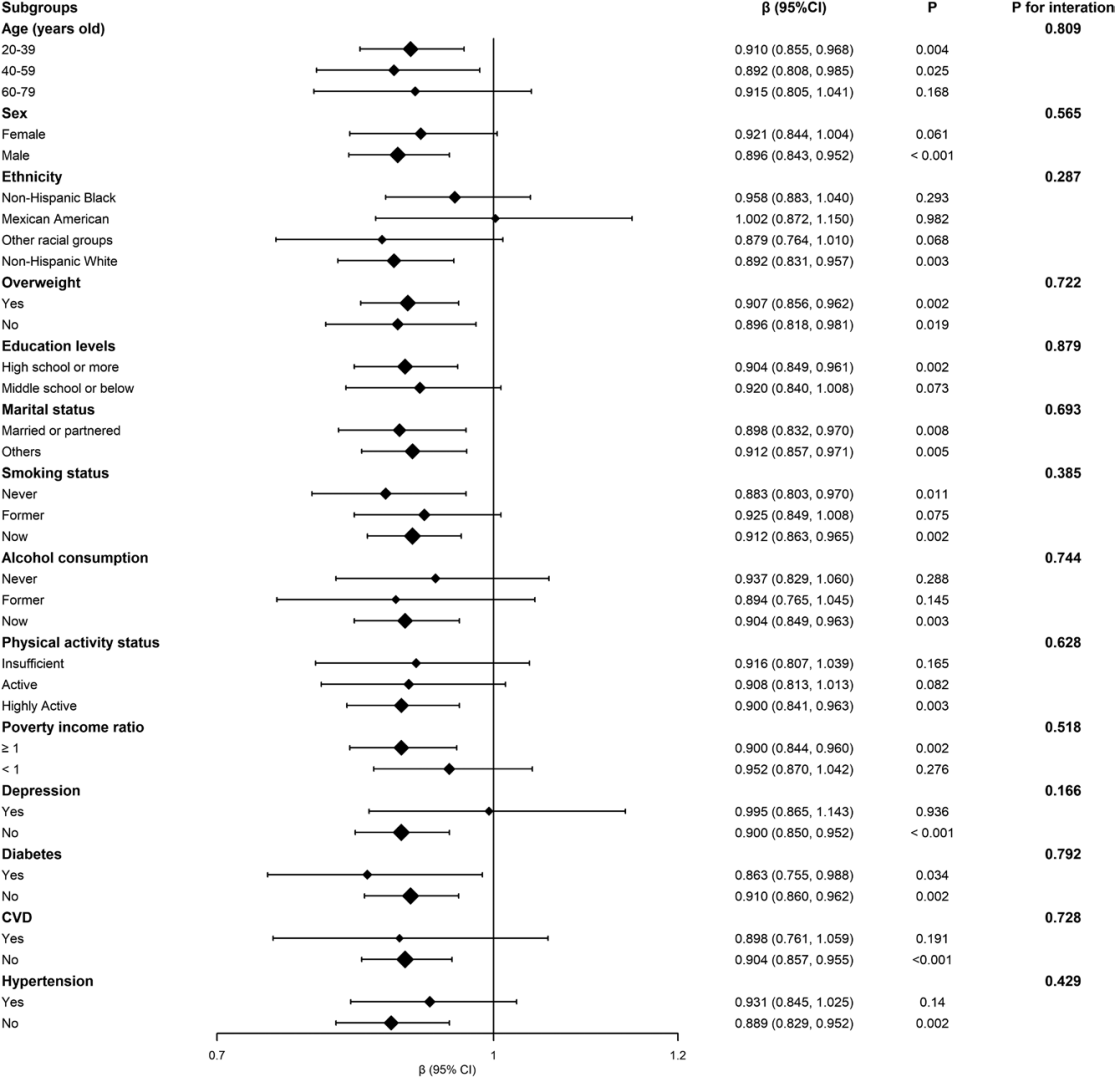
Subgroup analysis for the association between flavone intake and PhenoAgeAccel. CI = confidence interval.

Subgroup analyses demonstrated consistent inverse associations between log-transformed dietary flavone intake and PhenoAgeAccel across most demographic and health strata (Fig. 3). The association remained significant in adults aged 20–39 (*β *= 0.910; 95% CI: 0.855–0.968) and 40–59 years (*β *= 0.892; 95% CI: 0.808–0.985), but not in those aged 60–79 years. However, no significant interaction by age was detected (*P* for interaction = .809). Similar associations were observed across sex, ethnicity, BMI, education level, marital status, smoking and alcohol use, physical activity, and poverty status (all *P* for interaction > .05). The association also persisted among participants with or without diabetes, hypertension, or cardiovascular disease. Among individuals without depression, the association remained significant (*β* = 0.900; 95% CI: 0.850–0.952), whereas it was non-significant in those with depression (*P* = .936). However, this interaction was not statistically significant (*P* for interaction = .166). Overall, no significant effect modification was noted, underscoring the robustness of the association.

### 3.4. Association between intakes of two flavone subclasses and PhenoAgeAccel

As shown in Table 3, multivariable-adjusted analyses unveiled that higher intake of apigenin and luteolin were independently and significantly associated with lower PhenoAgeAccel. For apigenin, participants in the third (Q3) and highest (Q4) quartiles exhibited significantly lower PhenoAgeAccel than the lowest quartile (Q1), with *β* = 0.652 (95% CI: 0.530–0.804; *P* < .001) and *β* = 0.647 (95% CI: 0.499–0.839; *P* = .002), respectively. The continuous model also showed a significant inverse association when apigenin intake was analyzed continuously (*β* = 0.506; 95% CI: 0.285–0.896; *P* = .021), with a clear trend across quartiles (*P* for trend < 0.001). For luteolin, the highest quartile (Q4) also exhibited a significant inverse association compared with Q1 (*β* = 0.736; 95% CI: 0.584–0.927; *P* = .011), and a significant overall trend was observed across quartiles (*P* for trend = 0.008). The continuous model displayed a borderline association (*β* = 0.767; 95% CI: 0.586–1.004; *P* = .053).

**Table 3:** Multivariable-adjusted associations of apigenin and luteolin intake with phenotypic age acceleration.

Variables		β (95% CI)	*P* value	*P* for trend
Apigenin	Categories			<.001
	Q1	ref		
	Q2	0.858 (0.642, 1.147)	.287	
	Q3	0.652 (0.530, 0.804)	<.001	
	Q4	0.647 (0.499, 0.839)	.002	
	Continuous	0.506 (0.285, 0.896)	.021	
Luteolin	Categories			.008
	Q1	ref		
	Q2	0.919 (0.730, 1.159)	.461	
	Q3	0.788 (0.621, 1.001)	.050	
	Q4	0.736 (0.584, 0.927)	.011	
	Continuous	0.767 (0.586, 1.004)	.053	
Synergistic effects				
Apigenin × Luteolin	Continuous	1.446 (0.933, 2.242)	.095	
Apigenin	Continuous	0.357 (0.189, 0.677)	.003	
Luteolin	Continuous	0.876 (0.701, 1.094)	.231	

Apigenin and luteolin intake were log-transformed and analyzed as a continuous variable in a regression model.

Model was adjusted for age, sex, body mass index, ethnicity, poverty status, education status, physical activity status, marital status, smoking status, alcohol consumption, energy intake, depression, cardiovascular disease, hypertension, and diabetes.

To assess potential synergistic effects between apigenin and luteolin, we specifically tested the continuous interaction term (apigenin × luteolin, both log-transformed) in weighted multivariable logistic regression models.

CI = confidence interval, Q1–Q4 = quartiles 1–4.

The interaction between apigenin and luteolin intake, assessed using a continuous model, suggested a marginally non-significant positive association (*β *= 1.446, 95% CI: 0.933, 2.242, *P* = .095). In RCS analyses, a significant non-linear association was observed between dietary intakes of apigenin and luteolin and PhenoAgeAccel (*P* for non-linearity < .001) (Fig. 2).

## 4. Discussion

In this large, nationally representative sample of US adults, higher dietary intake of flavones was significantly associated with reduced PhenoAgeAccel, a validated biomarker of biological aging. This inverse association was robust even after adjustment for a wide range of sociodemographic, lifestyle, and health-related covariates and was consistent across key population subgroups. Furthermore, the 2 principal flavones, apigenin and luteolin, were independently associated with lower PhenoAgeAccel, with evidence of non-linear dose–response relationships. These results underscore the potential role of dietary flavones as modifiable factors for mitigating biological aging, though causal claims require verification through longitudinal or experimental designs.

Our results are consistent with those of Samieri et al, who found that higher midlife intake of flavones was associated with increased odds of healthy aging in women, defined as survival to age 70 or older without major chronic diseases or substantial cognitive, physical, or mental impairments.^[22]^ Extending this evidence, our study emphasizes the relationship between flavonoid intake and biological aging through the lens of PhenoAgeAccel. Unlike chronological age-based metrics, PhenoAgeAccel accurately captures aging-related physiological decline and offers a refined framework for evaluating the potential anti-aging effects of specific dietary components such as flavones. Additionally, earlier studies have shown that higher total flavonoid intake is associated with lower biological age across multiple organ systems, including the whole body, heart, and liver.^[35,36]^ However, these studies did not investigate the specific roles of flavonoid subclasses such as apigenin and luteolin. Moreover, their use of the simple difference between biological and chronological age (∆age) may have introduced bias due to its intrinsic correlation with chronological age. By contrast, our use of PhenoAgeAccel, defined as the residual from a regression of phenotypic age on chronological age, minimizes age-related confounding, offering a more stable and informative measure of biological aging. Our analysis showed that higher intakes of apigenin and luteolin were significantly associated with lower PhenoAgeAccel, suggesting that these specific subclasses may exert targeted effects on aging biology. These results provide valuable evidence that dietary components can influence aging trajectories. Given the global increase in aging populations and age-related diseases, identifying modifiable nutritional factors such as flavone intake could inform public health strategies aimed at delaying biological aging and promoting healthspan. Further research is warranted to replicate these findings in diverse populations and to clarify potential dose–response relationships and long-term outcomes.

Beyond observational associations, accumulating experimental evidence supports the biological plausibility of the anti-aging effects of apigenin and luteolin. Apigenin has been shown to counteract cellular and physiological aging through multiple mechanisms. It enhances dermal fibroblast viability, reduces UVA-induced cellular senescence, and inhibits matrix metalloproteinase-1 (MMP-1), thereby preserving extracellular matrix integrity and skin elasticity.^[37]^ In vascular aging models, apigenin improves endothelial function by increasing nitric oxide (NO) bioavailability and reducing arterial stiffness, oxidative stress, and inflammation.^[38]^ In mouse models, it delays systemic aging by activating the nuclear factor erythroid 2-related factor 2 (Nrf2) signaling pathway and upregulating antioxidant genes such as heme oxygenase-1 (HO-1) and NAD(P)H quinone dehydrogenase 1 (NQO1), while boosting endogenous antioxidant enzyme activity.^[39]^ At the cellular level, apigenin regulates the SIRT1–NAD⁺–CD38 axis, promoting DNA repair, reducing oxidative stress, and maintaining mitochondrial homeostasis.^[40]^ Luteolin also exhibits potent anti-aging effects, particularly through neuroprotective and systemic mechanisms. As a strong activator of SIRT1, luteolin helps maintain organelle integrity, including mitochondria, endoplasmic reticulum, Golgi apparatus, and lysosomes, during cellular aging processes.^[41]^ In D-galactose-induced brain aging models, luteolin improved cognitive function, restored cholinergic balance, reduced hippocampal oxidative stress and neuroinflammation, and enhanced SIRT1 expression.^[42]^ It also protects against vascular calcification, a hallmark of vascular aging, by lowering reactive oxygen species (ROS) and apoptosis, while enhancing autophagy through the SIRT1/C-X-C chemokine receptor type 4 (CXCR4) signaling pathway.^[43]^ Together, apigenin and luteolin exert their anti-aging effects through overlapping molecular pathways, including SIRT1 activation, oxidative stress suppression, anti-inflammatory activity, and cellular senescence regulation. These mechanistic insights reinforce our study’s findings and support the therapeutic potential of flavone-rich diets in promoting healthy biological aging.

This study benefits from several strengths, including the use of the NHANES dataset, which employs a complex, multistage, stratified sampling design to ensure national representativeness. The application of PhenoAgeAccel provides a validated and biologically meaningful measure of aging, independent of chronological age. Additionally, comprehensive adjustment for potential confounders, including sociodemographic, lifestyle, and clinical variables, enhances the credibility of our findings. Nevertheless, several limitations must be acknowledged. First, the cross-sectional design precludes causal inference, and residual confounding from unmeasured factors like supplement use or overall dietary patterns cannot be ruled out. Second, the exclusion of 19,094 participants (63.8%) was indeed substantial and warrants careful consideration of potential selection bias. The characteristics of included and excluded participants were shown in Table S2, Supplemental Digital Content, https://links.lww.com/MD/Q885. Third, dietary intake was assessed using 24-h dietary recall, which may be prone to recall bias and may not capture habitual dietary patterns. Fourth, the generalizability of the findings is limited to the US population, and validation in other populations is necessary. Fifth, spearman correlation analysis revealed a moderate positive association between apigenin and luteolin (ρ = 0.443). In the multivariable regression model, when both compounds were included simultaneously, Apigenin demonstrated an independent protective effect (OR = 0.357, *P *= .003), whereas Luteolin’s association became non-significant (OR = 0.876, *P* = .231), suggesting its effect might be influenced by apigenin or affected by collinearity issues.

## 12. Conclusion

In conclusion, our cross-sectional analysis of a nationally representative US adult population indicates that higher dietary intake of flavones, particularly apigenin and luteolin, may be associated with lower PhenoAgeAccel. The observed associations, which persisted after comprehensive covariate adjustment, are biologically plausible given the established antioxidant and anti-inflammatory properties of these compounds. While these findings merit further investigation in longitudinal and intervention studies, they tentatively suggest that increasing consumption of flavone-rich foods could be explored as a potential dietary approach to support healthy aging. Future studies should combine rigorously dosed RCTs with biomarker-enhanced longitudinal designs to establish causality and elucidate mechanisms.

## Acknowledgments

The authors are grateful to the National Health and Nutrition Examination Survey (NHANES) team for providing the data.

## Author contributions

**Conceptualization:** Wujian Peng, Peijia Liu.

**Data curation:** Chang Liu, Shuyan Tian.

**Formal analysis:** Xiaoqiao Wang, Guixia Li.

**Funding acquisition:** Wujian Peng, Peijia Liu.

**Methodology:** Shuyan Tian, Wujian Peng.

**Supervision:** Guixia Li, Wujian Peng, Peijia Liu.

**Writing – original draft:** Xiaoqiao Wang.

**Writing – review & editing:** Guixia Li, Peijia Liu.

## Supplementary Material
