# Supplementary material for: Associations of dietary flavones, particularly apigenin and luteolin, with phenotypic age acceleration: A cross-sectional study using NHANES data
Source: Medicine (Baltimore). 2025 Dec 12;104(50):e46520. doi: 10.1097/MD.0000000000046520 (PMC12708091; doi:10.1097/MD.0000000000046520)
Supplement: Supplementary file 1 [file medi-104-e46520-s001.pdf]

Method S1

The phenotypic age was calculated by nine parameters including albumin (g/L), creatinine (μmol/L), glucose (mmol/L), C-reactive protein (CPR, mg/dL), lymphocyte percent (%), mean red cell volume (fL), red cell distribution width (%), alkaline phosphatase (u/L), and white blood cell count (1000 cells/uL). The detailed equation was illustrated as follows:

$$\text{Phenotypic Age} = 141.5 + \frac{\ln\left[-0.0553 \times \ln\left(1 - xb\right)\right]}{0.09165}$$

where

$$\begin{aligned} xb = {} & -0.0336 \times albumin + 0.0095 \times creatinine + 0.1953 \times glucose \\ & + 0.0954 \times \ln(CRP) - 0.0120 \times lymphocyte\ percent \\ & + 0.0268 \times mean\ red\ cell\ volume \\ & + 0.3306 \times red\ cell\ distribution\ width \\ & + 0.0019 \times alkaline\ phosphatase + 0.0554 \times white\ blood\ cell\ count \\ & + 0.0804 \times chronological\ age \end{aligned}$$

Table S1. Association of Dietary Flavones Intake with PhenoAgeAccel among US Adults: Sensitivity Analysis Including Individuals with Cancer, Pregnant Women, and Renal Dysfunction (Serum Creatinine >442 μmol/L)

Flavones	Model 1			Model 2			Model 3	
	β (95%CI)	*P* value		β (95%CI)	*P* value		β (95%CI)	*P* value
Continuous	0.864 (0.823, 0.907)	< 0.001	Continuous	0.866 (0.824, 0.909)	< 0.001	Continuous	0.912 (0.866, 0.961)	0.001
Categories			Categories			Categories		
Q1	Ref		Q1	Ref		Q1	Ref	
Q2	0.718 (0.605, 0.851)	< 0.001	Q2	0.730 (0.614, 0.867)	< 0.001	Q2	0.827 (0.661, 1.033)	0.091
Q3	0.592 (0.481, 0.730)	< 0.001	Q3	0.599 (0.487, 0.738)	< 0.001	Q3	0.751 (0.610, 0.924)	0.009
Q4	0.520 (0.413, 0.655)	< 0.001	Q4	0.521 (0.415, 0.655)	< 0.001	Q4	0.674 (0.538, 0.846)	0.001
P for trend	< 0.001			< 0.001			< 0.001	

Notes: Flavones intake was log-transformed and analyzed as a continuous variable in a regression model.

Abbreviations: CI, confidence interval; PhenoAgeAccel, phenotypic age acceleration; Q1–Q4, quartiles 1–4

Model 1 did not include any covariate.

Model 2 was adjusted for age and sex.

Model 3 was adjusted for age, sex, body mass index, ethnicity, poverty status, education status, physical activity status, marital status, smoking status, alcohol consumption, energy intake, depression, cardiovascular disease, hypertension, and diabete.

Table S2 Characteristics of participants included and excluded in the study (age > 19 years)

Variables	Participants included *n* = 10,847	Participants excluded n = 6,184	*P* value
Sex, %			< 0.001
Male	5592 (51.6)	2675 (43.2)	
Age, years (mean (SD))	50.3 (17.0)	50.7 (19.2)	0.259
BMI, kg/m^2 (mean (SD))	29.4 (6.8)	29.2 (7.2)	0.104
Ethnicity, %			< 0.001
Non-Hispanic Black	2178 (20.1)	1341 (21.7)	
Mexican American	1856 (17.1)	925 (15.0)	
Other racial groups	1989 (18.3)	1355 (21.9)	
Non-Hispanic White	4823 (44.5)	2564 (41.5)	
Education levels, %			< 0.001
Middle school or lower	2822 (27.8)	1727 (30.9)	
Marital status, %			< 0.001
Married or partnered	6580 (60.6)	3533 (57.1)	
PIR, %			0.014
< 1	1972 (20.0)	1167 (21.7)	
Smoking status, %			0.078
Former	2699 (24.9)	1455 (23.5)	
Never	5872 (54.1)	3449 (55.8)	
Now	2274 (21.0)	1276 (20.6)	
Drinking status, %			0.003
Former	1475 (15.2)	622 (13.3)	
Never	1294 (13.3)	681 (14.5)	
Now	6930 (71.5)	3381 (72.2)	
Physical activity status, %			0.464
Insufficient	1426 (17.9)	789 (18.8)	
Active	1329 (16.7)	681 (16.2)	
Highly active	5190 (65.3)	2730 (65.0)	
Energy intake, kcal/d (mean (SD))	2113 (1021)	2015 (972)	< 0.001
Depression status, %			0.085
Clinically significant depression	941 (9.2)	509 (10.1)	
CVD, %			< 0.001
Yes	1190 (11.0)	832 (13.5)	
Hypertension, %			0.858
Yes	4700 (43.3)	2690 (43.5)	
Diabetes, %			0.024
Yes	2086 (19.2)	1242 (20.7)	

Abbreviations: PIR, poverty income ratio; BMI, body mass index; CVD, cardiovascular disease.
